# Supplementary material for: On the nanoscale structural evolution of solid discharge products in lithium-sulfur batteries using operando scattering
Source: Nat Commun. 2022 Oct 24;13:6326. doi: 10.1038/s41467-022-33931-4 (PMC9592616; doi:10.1038/s41467-022-33931-4)
Supplement: Supplementary file 1 — Supplementary Information [file 41467_2022_33931_MOESM1_ESM.pdf]

Supplementary Information

On the nanoscale structural evolution of solid discharge products in Lithium-Sulfur batteries using operando scattering

Christian Prehal[1, *], Jean-Marc von Mentlen[1], Sara Drvarič Talian[2], Alen Vizintin[2], Robert Dominko[2, 3], Heinz Amenitsch[4], Lionel Porcar[5], Stefan A. Freunberger[6, *], Vanessa Wood[1, *]

1. Department of Information Technology and Electrical Engineering, ETH Zürich, Gloriastrasse 35, 8092 Zürich, Switzerland
2. Department of Materials Chemistry, National Institute of Chemistry, Hajdrihova 19, 1000 Ljubljana, Slovenia
3. Faculty of Chemistry and Chemical Technology University of Ljubljana, Večna pot 113, 1000 Ljubljana, Slovenia
4. Institute for Inorganic Chemistry, Graz University of Technology, Stremayrgasse 9, 8010 Graz, Austria
5. Institut Laue–Langevin, 71 Avenue des Martyrs, Grenoble, 38042, France
6. IST Austria (Institute of Science and Technology Austria), Am Campus 1, 3400 Klosterneuburg, Austria

* Corresponding authors' e-mail: cprehal@ethz.ch, stefan.freunberger@ist.ac.at, vwood@ethz.ch

Supplementary Figures

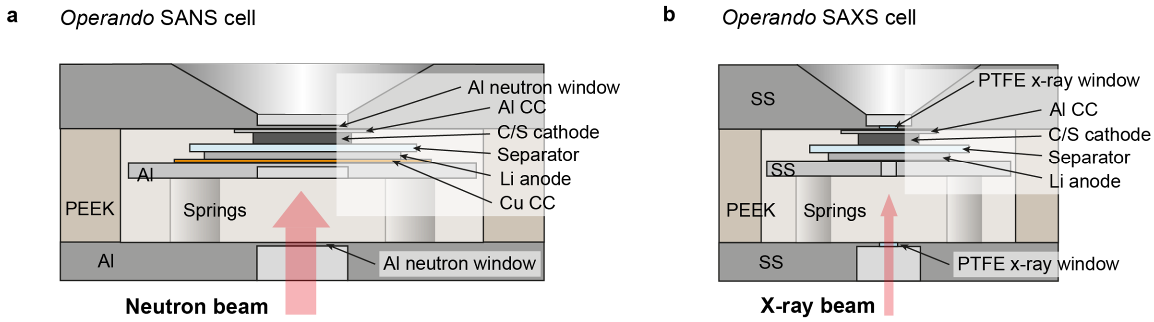

Supplementary Figure 1: Schematics of operando SANS cell **(a)** and operando SAXS / WAXS cell **(b)**. The design looks similar, only the Aluminum windows of the SANS cell are replaced by thin PTFE windows in the SAXS cell. Note that the Aluminum windows are only cavities in the upper and bottom cell parts. Given the larger diameter of the neutron beam (10mm) the SANS cell is slightly larger in diameter.

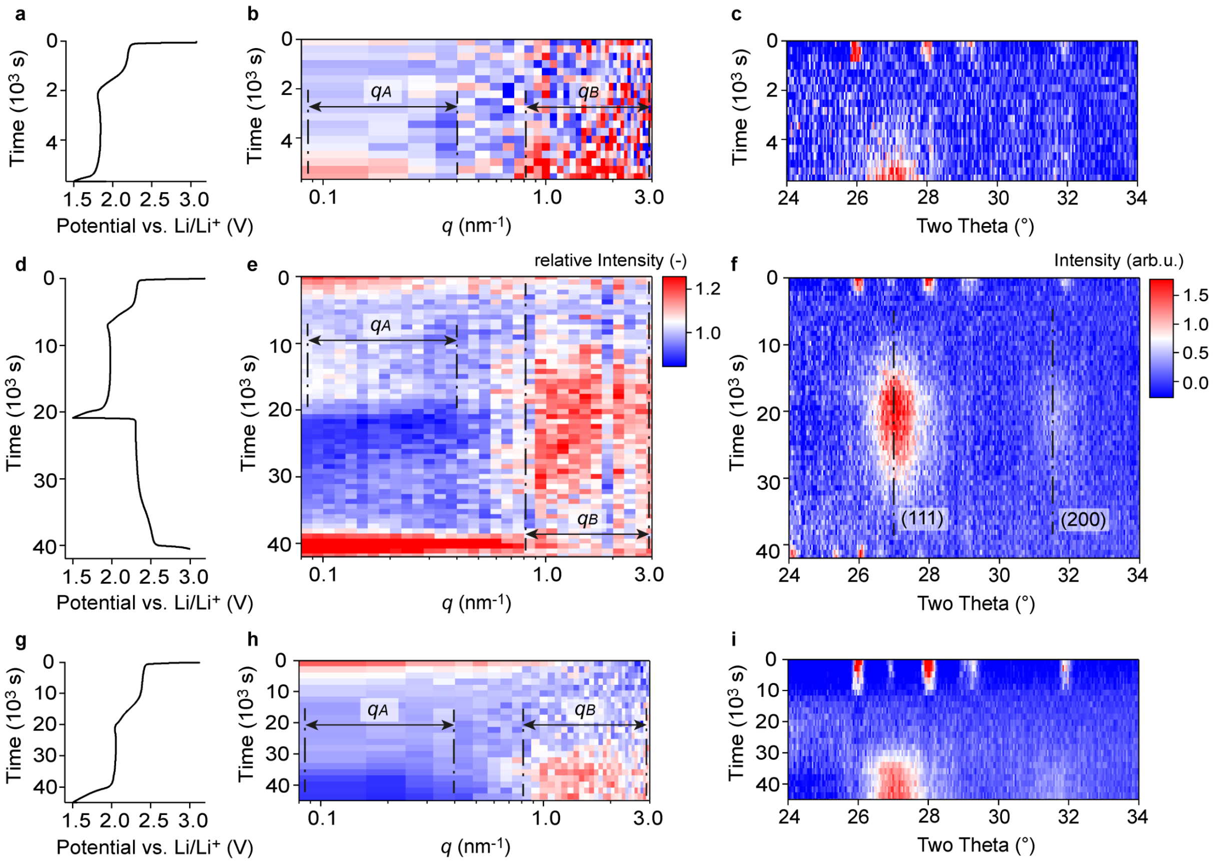

Supplementary Figure 2: Operando SAXS / WAXS during galvanostatic discharge / charge of a ENSACO350G/S composite cathode and a 1 M LiTFSI TEGDME:DOL (1:1) electrolyte at 1.23 mA cm^{-2} (**a-c**), 0.39 mA cm^{-2} (**d-f**) and 0.12 mA cm^{-2} (**g-i**). The SAXS/WAXS data were recorded on a laboratory SAXS facility (SAXSpoint 2.0, Anton Paar), resulting in a significantly higher signal-to-noise ratio as compared to the Synchrotron data. **a**, Potential vs. Li/Li$^+$ as a function of time during galvanostatic discharge at 1.23 mA cm^{-2}. **b**, The corresponding relative SAXS intensity change as a function of time and scattering vector length q. **c**, WAXS intensity as a function of time and scattering angle Two Theta. **d-e** and **h-i** show the same data for a full galvanostatic discharge / charge cycle at 0.39 mA cm^{-2} (**d-e**) and galvanostatic discharge at 0.12 mA cm^{-2} (**h-i**). The SAXS/WAXS data verify the features observed in Fig. 2 in an alternative Li-S system. The high-q (q_B) SAXS intensity maximum at the end of discharge is present at all rates; the low-q (q_A) SAXS intensity maximum is more pronounced at higher applied currents. The Scherrer crystallite sizes at 1.23 mA cm^{-2}, 0.39 mA cm^{-2} and 0.12 mA cm^{-2} correspond to 6.5 nm, 6.7 nm and 6.0 nm obtained from a Lorentzian peak fit and the Scherrer equation. This is practically identical to the values shown in Fig. 2. The sharp peaks in **c, f, i** correspond to the diffraction peaks of the relatively large sulfur crystallites.

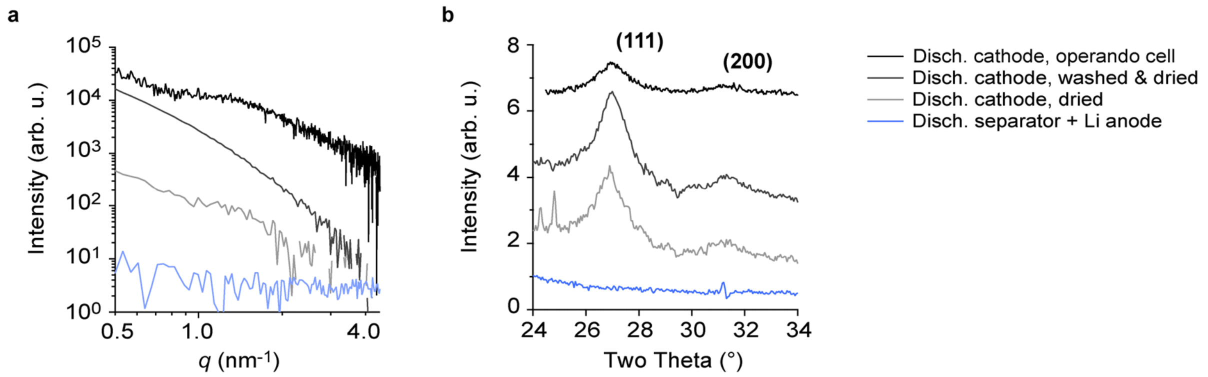

Supplementary Figure 3: SAXS/WAXS intensities versus scattering vector length q (**a**) and scattering angle (**b**) for the discharged cathode in the *operando* cell. The black solid line shows the equivalent to the reduced SAXS intensity after discharge in Fig. 5a, the discharged cathode after washing with 1-Methoxy-2-(2-methoxyethoxy)ethane (diglyme) and subsequent drying under vacuum (dark grey solid line) and the discharged cathode without washing, but with drying under vacuum (grey solid line). The blue solid line shows the SAXS/WAXS intensities of separator and Li metal after discharge. The absence of any SAXS feature and the Li_2S diffraction peaks gives evidence that the recorded structural changes take place in the cathode only.

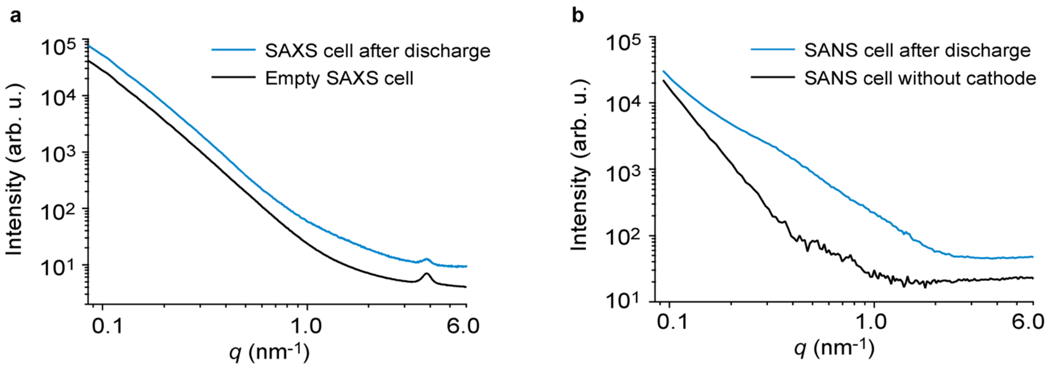

Supplementary Figure 4: a, SAXS intensities versus scattering vector length *q* of the SAXS cell after potentiostatic discharge at 2.0 V vs. Li/Li+ and the empty SAXS cell. This shows (together with the SAXS/WAXS intensity of separator and Li metal anode in Supplementary Fig. 3) that the intensity changes in Fig. 2 stem from the cathode only. **b**, SANS intensities versus scattering vector length q of the SANS cell after potentiostatic discharge at 2.0 V vs. Li/Li^+ and the SANS cell without cathode. The significant SANS intensity changes stem from nanostructures in the cathode only.

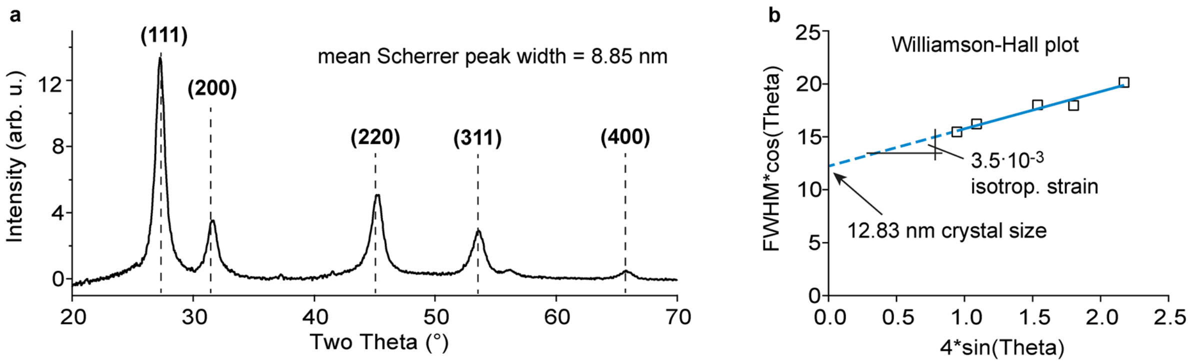

Supplementary Figure 5: a, X-ray diffraction intensity versus scattering angle of a fully galvanostatically discharged carbon black (KetjenBlack EC-600JD, ANR Technologies) / sulfur electrode (1:2 carbon/S mass-ratio) at 1.02 mA cm^{-2}. The mean crystallite size estimated from the peak widths and the Scherrer equation is 8.85 nm. **b**, Williamson-Hall plot[1] showing the significant isotropic strain of $3.5*10^{-3}$ in the Li_2S nanocrystals. This results in significant peak broadening and an estimated crystallite size of 12.83 nm, which is similar to values in literature[2,3]. We conclude that the actual crystallite size of Li_2S formed during electrochemical discharge can be ~ 50% larger than the Scherrer equation suggests.

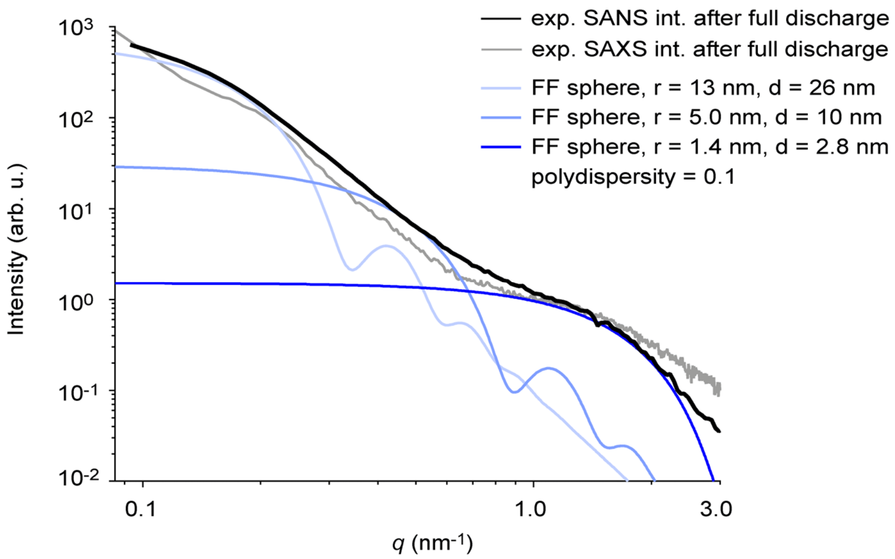

Supplementary Figure 6: The blue curves correspond to the analytical SAXS/SANS formfactors of spheres with 10 % polydispersity and a mean diameter of 26 nm, ~ 10 nm and 2.8 nm and give an estimate about the size of possible real-space structures. For comparison we show the background-corrected SANS intensity versus scattering vector length q for the fully discharged KB cathode in the deuterated diglyme catholyte after subtracting the SANS intensity prior to discharge at open circuit voltage (OCV) (black solid line, details see methods). In grey we show the background-corrected SAXS intensity after full discharge. Deviations to the SANS intensity are caused by the significant contribution of the carbon black scattering. This contribution is minimized with SANS using deuterated electrolyte. The primary Li_2S crystallites with a size of ~ 10 nm (as obtained from the Li_2S WAXS diffraction peak width) are not present as individual, solid particles since the corresponding intensity shoulder is missing in the SAS regime. The high-q (1.5 nm^{-1}) and low-q (0.2 nm^{-1}) intensity shoulders correspond to solid particles / aggregates with a size around 2.8 nm and 26 nm, respectively.

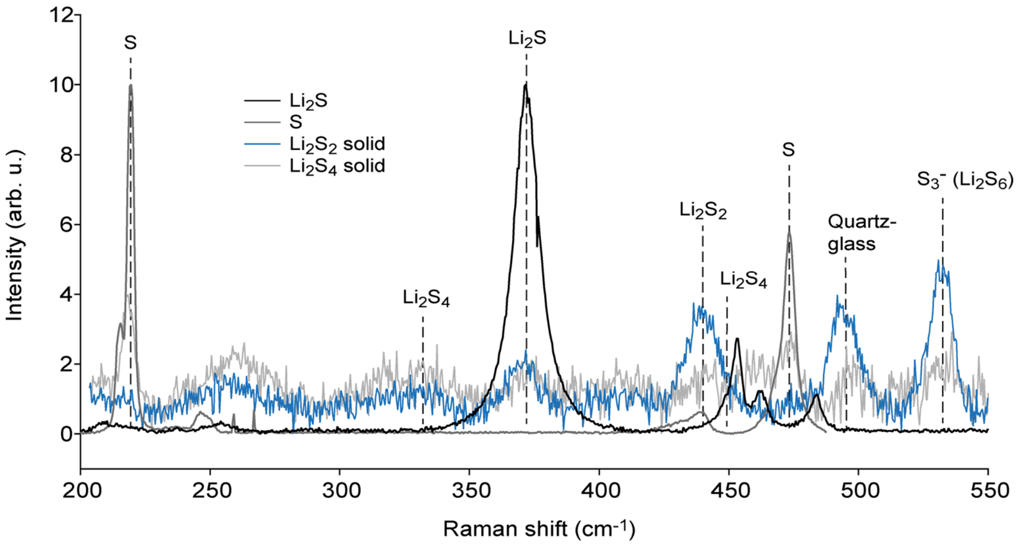

Supplementary Figure 7: Reference Raman spectra of commercial Li_2S, commercial S and nominal solid Li_2S_2 and solid Li_2S_4 powder. The Li_2S_2 and Li_2S_4 powders were prepared by mixing Li and S in the right stoichiometry in a solution of THF. After Li and S has dissolved/dispersed in the solvent, the liquid is dried under vacuum. The peak positions at 373 cm^{-1} (Li_2S), 440 cm^{-1} (Li_2S_2) and 534 cm^{-1} (S_3^-, Li_2S_6) are in line with literature [4-6]. The Raman spectrum of nominal Li_2S_2 shows that it consists of several components such as Li_2S, Li_2S_2 and Li_2S_6 (S_3^-). These components are similar to the components of the discharged electrode (Fig. 3c).

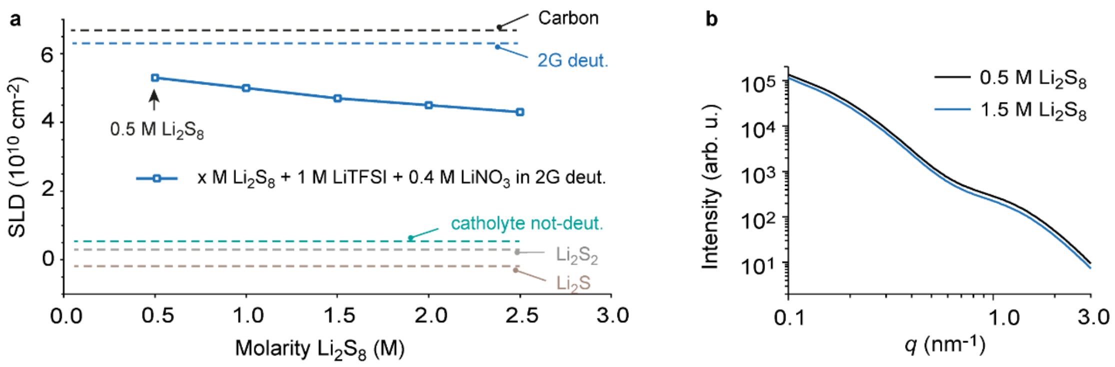

Supplementary Figure 8: a, Scattering length densities (SLDs) vs. Li_2S_8 molarity in the deuterated catholyte. The dashed lines indicate the SLD of other components in the system. **b**, Modelled SANS intensities for the Li_2S/Li_2S_2 structure at the end of discharge. Structural parameters are taken from Supplementary Table 2, only the catholyte SLDs are different. The black curve uses the SLD for 0.5 M Li_2S_8, the blue line the SLD for 1.5 M Li_2S_8. The SANS intensities are only shifted by a small constant factor.

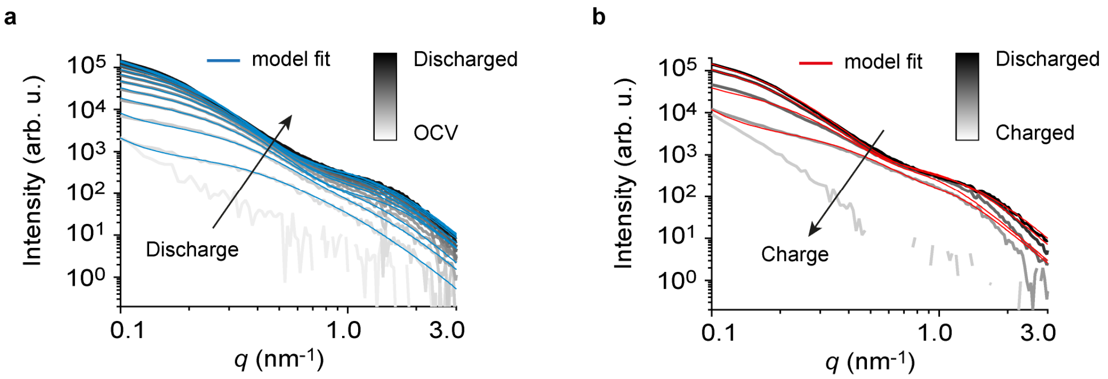

Supplementary Figure 9: a, SANS intensities versus scattering vector length q during potentioststatic discharge at 2.0 V vs. Li/Li+ (see Fig. 4b). Solid blue lines correspond to model fits using plurigaussian random fields. b, SANS intensities versus momentum transfer q during potentioststatic charge at 2.45 V vs. Li/Li+ (see Fig. 4b). Solid blue lines correspond to model fits using plurigaussian random fields. Fit parameters are shown in Supplementary Fig. 11.

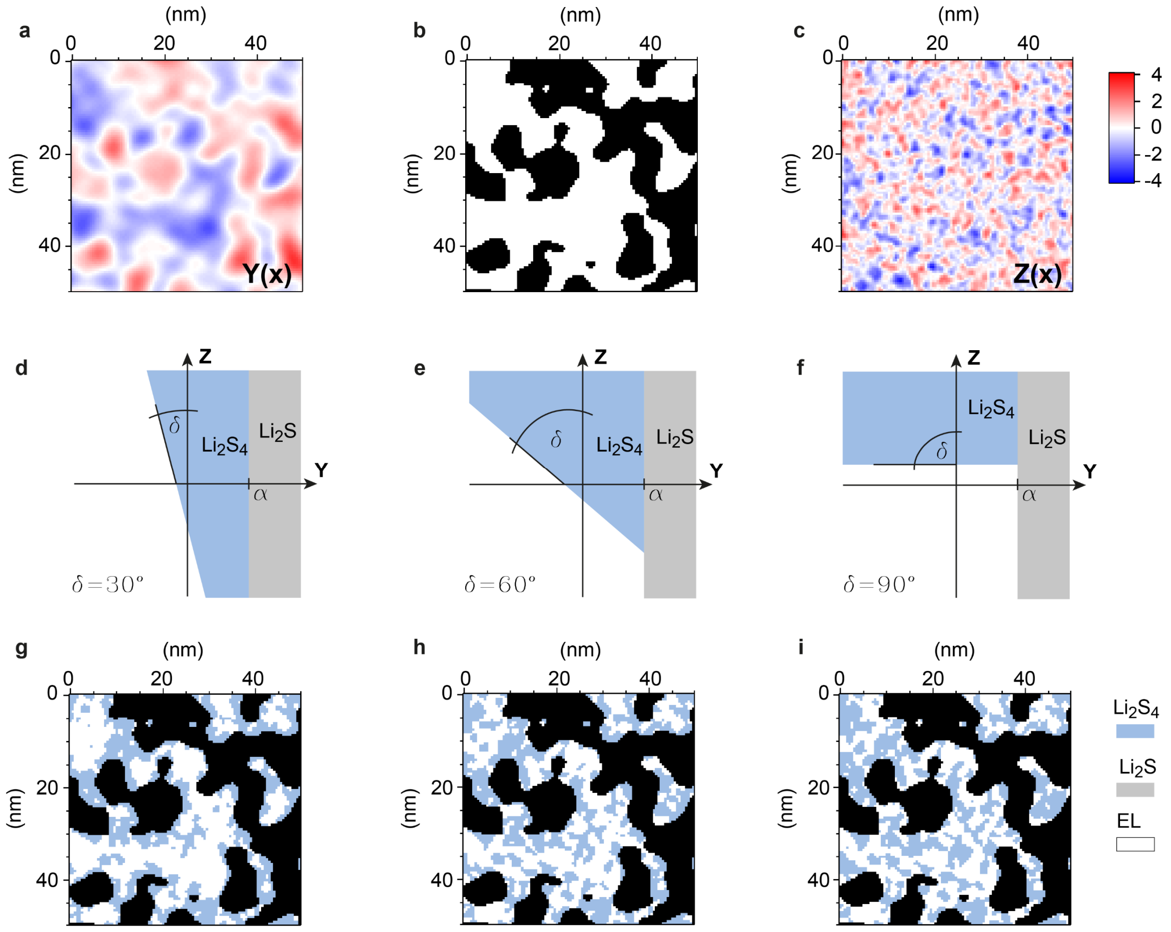

Supplementary Figure 10: Plurigaussian random field (PGRF), real space structure generation with the parameters shown in Supplementary Table 1, second column. Only the parameter δ is varied. **a**, Cross-section of 3D Gaussian random field $Y(\boldsymbol{x})$. **b**, Cross section of 3D Li_2S structure generated from $Y(\boldsymbol{x})$ using the threshold value α. **c**, Cross-section of 3D Gaussian random field $Z(\boldsymbol{x})$. **d-e**, Z-Y planes visualizing the threshold value dependencies of the two GRFs $Y(\boldsymbol{x})$ and $Z(\boldsymbol{x})$. The threshold value α to generate the Li_2S structure is indicated on the horizontal axis. The borderline between blue and white area determines whether the Li_2S_4 phase shows a strong correlation (**d**, $\delta \to 0°$) or no correlation (**f**, $\delta \to 90°$) to the Li_2S phase. The corresponding morphologies for $\delta = 30°$, $\delta = 60°$, and $\delta = 90°$ are shown in **g**, **h** and **i**, respectively.

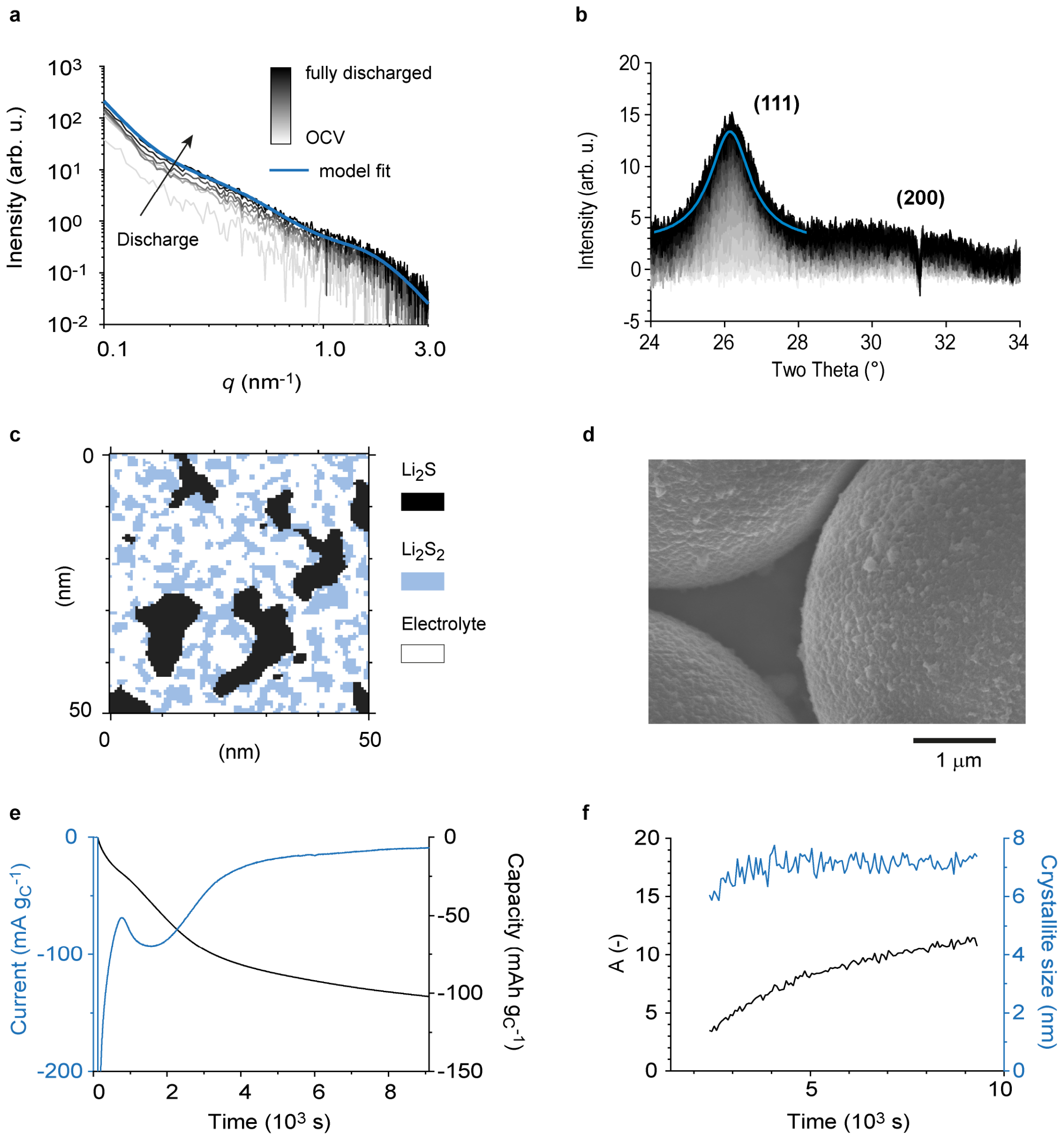

Supplementary Figure 11: Operando SAXS / WAXS and plurigaussian random field model for potentiostatic discharge with a Glassy Carbon bead electrode and the 0.5 M Li_2S_8 / 2G catholyte as used in Fig. 1 - 2. **a**, reduced, background-corrected SAXS intensities versus scattering vector length q during potentiostatic discharge at 2.0 V vs. Li/Li$^+$ to a capacity of 102 mAh g_C^{-1}. The SAXS intensity prior to discharge (at OCV) was subtracted from averaged SAXS intensities. As the glassy carbon beads are much larger than the Li_2S / Li_2S_2 deposits any cross correlations are negligible in the SAXS intensities. The plurigaussian random field (PGRF) model fit is given in blue. Fit parameters are given in Supplementary Table 1. **b**, Background corrected WAXS intensities versus scattering angle during potentiostatic discharge at 2.0 V vs. Li/Li$^+$ to a capacity of 250 mAh g_C^{-1}. The (111) Li_2S diffraction peak is fitted using a Lorentz function. **c**, Cross section of the representative real space model generated via PGRFs and the input parameters obtained from the model fit in **a**. **d**, Scanning electron microscopy image showing the Li_2S/Li_2S_2 deposits on top of the large Glassy carbon beads (> 1 µm) after potentiostatic discharge. **e**, Specific current (blue) and specific capacity (black) versus time during potentiostatic discharge of the operando SAXS cell. Both current and capacity are normalized by the bare glassy carbon electrode mass. **f**, (111) diffraction peak height A (obtained from Lorentzian peak fit) and crystallite size (obtained from the (111) peak width and the Scherrer equation) as a function of time during potentiostatic discharge. The good fit quality in **a** and the similar structural results compared to the SANS fits (despite the different scattering length densities) indicate that the used Li_2S/Li_2S_2 model is of high accuracy.

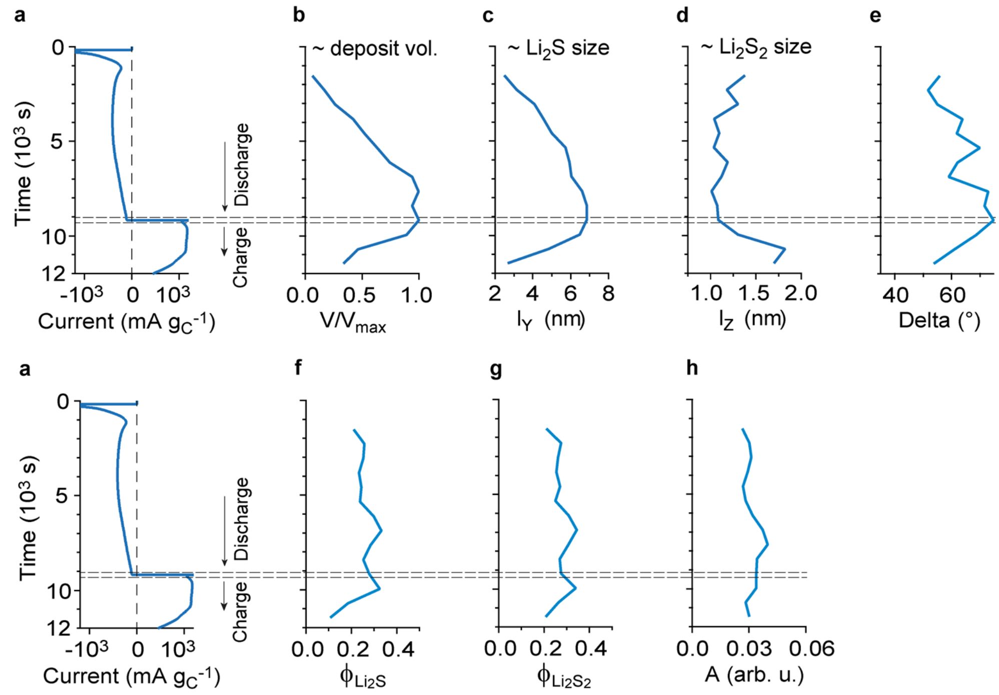

Supplementary Figure 12: Fit parameter as a function of time resulting from PGRF model fits of operando SANS data during potentiostatic discharge (2.0V vs Li/Li+) and charge (2.45 V vs. Li/Li+). Current vs. time (a), V/V_{max} vs. time. The constant K (Equation 4 in the main text) corresponds to 0.079. (b), l_Y vs. time (c), l_Z vs. time (d), Delta vs. time (e), ϕ_{Li2S} vs. time (f), ϕ_{Li2S2} vs. time. (h), Constant A vs. time.

Supplementary Tables

Supplementary Table 1 | Scattering length densities (SLDs) of the various phases. ρ_{e^-} is given in units of cm^{-2} and corresponds to the electron density times the classical electron radius (2.82×10^{-13} cm). The Li_2S_2 mass density, 1.62 $cm^3\ g^{-1}$, was taken from DFT simulations, calculating crystal structures of solid PSs[7]. For the catholyte, we assumed a Li_2S_8 concentration, higher than in the bulk liquid, resulting in an SLD of 1.1×10^{11} cm^{-2} for SAXS. For the SANS model fit we assumed that the deuterated catholyte SLD matches the carbon SLD. Supplementary Note 1 explains why this is valid. The carbon black skeleton density is lower than the graphite density due to the significant micropore content[8,9]. Catholyte corresponds to 1 M LiTFSI + 0.4 M $LiNO_3$ + 0.5 M Li_2S_8 in 2G(deuterated).

	Mass density ($g\ cm^{-3}$)	Molar mass ($g\ mol^{-1}$)	Molar volume ($cm^3\ mol^{-1}$)	SLD_{SAXS} (cm^{-2})	SLD_{SANS} (cm^{-2})
Li_2S	1.66	45.95	27.68	1.34×10^{11}	-0.21×10^{10}
Li_2S_2	1.62	78	48.15	1.335×10^{11}	0.24×10^{10}
S	2.00	32	16	1.69×10^{11}	1.11×10^{10}
2G (deuterated)	-	-	-	0.874×10^{11}	6.34×10^{10}
Carbon	2.0	12	5.85	1.74×10^{11}	6.67×10^{10}
Catholyte (deuterated)	-	-	-	0.98×10^{11}	5.3×10^{10}

Supplementary Table 2 | PGRF model fit results/input parameters. The scattering length densitiy (SLD) is given in units of cm^{-2}. For SAXS it corresponds to the electron density times the classical electron radius (2.82×10^{-13} cm).

	Carbon black electrode discharged (SANS model fit Fig. 4b)	Glassy Carbon electrode (SAXS model fit Figure S6a)
K	0.079	7.5
V / V_{max}	1	1
$\rho_{e-, EL}$ (cm^{-2})	6.67×10^{10}	1.10×10^{11}
$\rho_{e-, Li2S}$ (cm^{-2})	-0.21×10^{10}	1.34×10^{11}
$\rho_{e-, Li2S2}$ (cm^{-2})	0.24×10^{10}	1.335×10^{11}
l_Y (nm)	6.85	3.2
d_Y (nm)	102.75	32
l_Z (nm)	1.08	1.1
d_Z (nm)	6.95	4.6
ϕ_{Li2S}	0.28	0.15
ϕ_{Li2S2}	0.274	0.30
δ (°)	74.75	90
A	0.034	0.0048
γ	–4	–4.5

Supplementary Table 3 | Summary of investigated Li-S systems using SAXS/WAXS and SANS. KB is a high-surface area carbon black powder (1400 $m^2\ g_C^{-1}$), ENSACO another high-surface area carbon black powder, and GC are glassy carbon beads with a diameter of a few micrometers and a low surface area of 1.3 $m^2\ g_C^{-1}$.

	**Fig. 2**	**Fig. 4**	**Suppl. Fig. 10**	**Suppl. Fig. 2a**	**Suppl. Fig. 2b**	**Suppl. Fig. 2c**
Discharge /charge	potentiostatic 2.0/2.45 V	potentiostatic 2.0/2.45 V	potentiostatic 2.0/2.45 V	galvanostatic 1.23 mA cm^{-2}	galvanostatic 0.39 mA cm^{-2}	galvanostatic 0.12 mA cm^{-2}
Method	SAXS/WAXS	SANS	SAXS/WAXS	SAXS/WAXS	SAXS/WAXS	SAXS/WAXS
Cathode	KB	KB	GC	ENSACO/S 1:2	ENSACO/S 1:2	ENSACO/S 1:2
Catholyte/ Electrolyte	0.5 M Li_2S_8 + 1 M LiTFSI + 0.4 M $LiNO_3$ in 2G	0.5 M Li_2S_8 + 1 M LiTFSI + 0.4 M $LiNO_3$ in 2G deuterated	0.5 M Li_2S_8 + 1 M LiTFSI + 0.4 M $LiNO_3$ in 2G	1 M LiTFSI + 0.1 M $LiNO_3$ in 2G	1 M LiTFSI + 0.1 M $LiNO_3$ in 2G	1 M LiTFSI + 0.1 M $LiNO_3$ in 2G
E/S ratio ($\mu L\ mg_S^{-1}$)	7.81	7.81	7.81	156.25	143.44	162.04
S mass (mg)	7.68	25.6	7.68	1.12	1.22	1.08
C mass (mg)	0.72	5.13	4.42	0.56	0.61	0.53
Cathode diameteter (mm)	7	13	7	8	8	8
S mass loading (g cm^{-2})	19.96	19.29	19.96	2.23	2.43	2.15
Li anode purity	≥ 99.9 %	≥ 99.9 %	≥ 99.9 %	≥ 99.9 %	≥ 99.9 %	≥ 99.9 %
Li anode thickness (mm)	0.75	0.75	0.75	0.75	0.75	0.75
Li anode diameter (mm)	16	22	16	16	16	16

Supplementary Notes

Supplementary Note 1: electrolyte to sulfur (E/S) ratios and sulfur amount in catholytes

The E/S ratio in the 0.5 M Li2S8 catholyte is calculated using the following equation

$$\frac{E}{S}\,(\mu\mathrm{L\ mg^{-1}}) = \frac{1}{\mathrm{Molarity\ (mol\ L^{-1})} * M_{\mathrm{S8}}(\mathrm{g\ mol^{-1}})} * 10^{-6}(\mu\mathrm{L\ L^{-1}}) * 10^{3}(g\ mg^{-1}) \tag{S1}$$

The S mass in the catholyte then corresponds to a theoretical mass loading of

$$m_S(mg\ cm^{-2}) = \frac{\mathrm{Molarity\ (mol\ L^{-1})} * M_{\mathrm{S8}}(\mathrm{g\ mol^{-1}}) * V_{elyte}(\mathrm{L})}{(d_{\mathrm{el}}/2)^2 * \pi\ (\mathrm{cm^2})} * 10^{-3}(\mathrm{mg\ g^{-1}}) \tag{S2}$$

The calculated values are given in Supplementary Table 3.

Supplementary Note 2: scattering length density

The scattering length density (SLD) of the deuterated catholyte is 5.3e+10 cm^{-2}, which is a little smaller than the SLD of the carbon (Supplementary Fig. 8a). To estimate the intensity contribution let's assume a two-phase system, like carbon black in deuterated electrolyte. In such system, the SANS intensity scales with the square of the SLD difference (ΔSLD^2) between the two phases. The ΔSLD^2 correspond to 1.86e+20 cm^{-4} for carbon and catholyte and to 3.03e+21 cm^{-4} for Li_2S and catholyte. This means that the SANS scattering contribution of carbon is only about 6 % of the scattering contribution of solid Li_2S. Since the small remaining carbon scattering background is subtracted from all measured SANS intensities prior to data fitting in Fig. 4b, c (see methods, background subtraction), any remaining error could only stem from second-order effects like cross-correlations between the carbon and Li_2S/Li_2S_2 structure. Such error would be even smaller than the mentioned 6 % of the SANS intensity.

The blue data points in Supplementary Fig. 8a show the catholyte SLD for different polysulfide (Li_2S_8) concentrations. Starting from the 0.5 M Li_2S_8 concentration with an SLD of 5.3e+10 cm^{-2}, the SLD would decrease by a few %, when the Li_2S_8 concentration is increased. Even if the polysulfide concentration would increase by 200 % (from 0.5M to 1.5 M), the effect on the SANS intensity is only in the range of a few % (see equations 5 and 6 in the main text). Modeled SANS intensities with the same Li_2S/Li_2S_2 structure but different polysulfide concentrations in the catholyte confirm this (Supplementary Fig. 8b).

Overall, effects related to possible polysulfide concentration changes or errors related to our SLD assumptions are negligibly small compared to the large SANS intensity changes, which increases by two orders of magnitude upon solid Li_2S/Li_2S_2 formation (see Fig. 4b).

Supplementary References

1 Nath, D., Singh, F. & Das, R. X-ray diffraction analysis by Williamson-Hall, Halder-Wagner and size-strain plot methods of CdSe nanoparticles- a comparative study. *Mater. Chem. Phys.* **239**, 122021, (2020).

2 Li, Z., Zhou, Y., Wang, Y. & Lu, Y.-C. Solvent-Mediated Li2S Electrodeposition: A Critical Manipulator in Lithium–Sulfur Batteries. *Advanced Energy Materials* **9**, 1802207, (2019).

3 Drvarič Talian, S. *et al.* Which Process Limits the Operation of a Li–S System? *Chemistry of Materials* **31**, 9012-9023, (2019).

4 Partovi-Azar, P., Kühne, T. D. & Kaghazchi, P. Evidence for the existence of Li2S2 clusters in lithium–sulfur batteries: ab initio Raman spectroscopy simulation. *Physical Chemistry Chemical Physics* **17**, 22009-22014, (2015).

5 Wu, H.-L., Huff, L. A. & Gewirth, A. A. In Situ Raman Spectroscopy of Sulfur Speciation in Lithium–Sulfur Batteries. *ACS Applied Materials & Interfaces* **7**, 1709-1719, (2015).

6 Yeon, J.-T., Jang, J.-Y., Han, J.-G., Cho, J., Lee, K. T. & Choi, N.-S. Raman Spectroscopic and X-ray Diffraction Studies of Sulfur Composite Electrodes during Discharge and Charge. *Journal of The Electrochemical Society* **159**, A1308-A1314, (2012).

7 Xiao, J. *et al.* Elaboration of Aggregated Polysulfide Phases: From Molecules to Large Clusters and Solid Phases. *Nano Letters* **19**, 7487-7493, (2019).

8 Bahadur, J., Melnichenko, Y. B., He, L., Contescu, C. I., Gallego, N. C. & Carmichael, J. R. SANS investigations of CO2 adsorption in microporous carbon. *Carbon* **95**, 535-544, (2015).

9 Prehal, C., Koczwara, C., Jäckel, N., Amenitsch, H., Presser, V. & Paris, O. A carbon nanopore model to quantify structure and kinetics of ion electrosorption with in situ small angle X-ray scattering. *Phys. Chem. Chem. Phys.* **19**, 15549, (2017).
